# Multicenter, Retrospective, observational study to evaluate the real-world use and effectiveness of a fixed-ratio combination of insulin degludec/liraglutide (IDegLira) in the Pakistani population with Type-2 diabetes (T2D)

**DOI:** 10.12669/pjms.41.5.11002

**Published:** 2025-05

**Authors:** Umar Yousaf Raja, Muhammad Umar Wahab, Fawad Ahmed Randhawa, Arshad Hussain, Abbas Raza, Saeed Ahmed Mahar, Osama Ishtiaq, Tejhmal Rehman, Faisal Masood Qureshi, Ibrar Ahmed

**Affiliations:** 1 Umar Yousaf Raja Shifa International Hospitals Ltd., Islamabad, Pakistan; 2 Muhammad Umar Wahab Umar Diabetes and Foot Care Centre Office 1, Islamabad, Pakistan; 3 Fawad Ahmed Randhawa Hameed Lateef Hospital, Lahore, Pakistan; 4 Arshad Hussain North-West General Hospital, Peshawar, Pakistan; 5 Abbas Raza National Hospital, Lahore, Pakistan; 6 Saeed Ahmed Mahar Mahar Medical Clinic, Karachi, Pakistan; 7 Osama Ishtiaq Al-Khaliq Hospital, Multan, Pakistán; 8 Tejhmal Rehman Shifa International Hospitals Ltd., Islamabad, Pakistan; 9 Faisal Masood Qureshi; 10 Ibrar Ahmed Lady Reading Hospital, Peshawar, Pakistan. Shifa International Hospitals Ltd., Islamabad, Pakistan

**Keywords:** Diabetes mellitus Type-II, HbA1C, IDeglira, Premix insulin

## Abstract

**Objective:**

To evaluate the real-world use and effectiveness of a fixed-ratio combination of insulin degludec/liraglutide (IDegLira) in the Pakistani population with type II diabetes (T2D).

**Methods:**

This multicenter, retrospective study collected real-world data from various public & private hospitals, and primary care clinics in Pakistan. The total duration of clinical data collection was six months (April and September 2022) from 183 diabetic patients across different centers in Pakistan. Patients who had received IDegLira in combination with basal/premixed insulin, OHAs, or both regimes (basal/premixed insulin and OHAs) for the treatment of Type-II diabetes (T2D) in the Pakistani population were analyzed.

**Results:**

After receiving IDegLira therapy for six months, mean HbA1c was reduced by -1.1%, mean body weight reduced by 1.2 kg, fasting blood glucose levels (mg/dl): 164.7 (±36.2) to 134.4 (±32.9)] and random blood sugar (mg/dl): 240.1 (±71.4) to 207.6 (±77.8)]. The mean IDegLira dose steps were 19.9 (±6.7) at initiation and 23.5 (±7.9) at follow-up. Overall, adverse events reported were generally related to gastrointestinal, and few were related to headache, fatigue, and hypoglycemia events.

**Conclusion:**

The values of HbA1c and body weight decreased for the patients after using iDeglira for six months. Therefore, the medicine had a positive impact on the outcome of the study.

## INTRODUCTION

As per International diabetes federation, global prevalence of diabetes mellitus among adults was 10.5% in 2021 and this number will surge by 783 million by 2045.^1^ It is imperative to manage the disease effectively for the prevention of the long-term complications of T2D, including cardiovascular diseases, nephropathy, and retinopathy.^2^ Insulin degludec/liraglutide (IDegLira) is a novel fixed-ratio combination of a long-acting basal insulin (insulin degludec) and a glucagon-like peptide-1 receptor agonist (GLP-1 RA) (as liraglutide). This combination has shown promise in improving glycemic control while minimizing the risk of hypoglycemia and weight gain, common adverse effects associated with insulin therapy.^2^ Recent studies have demonstrated the efficacy and safety of IDegLira in various populations, highlighting its potential as an effective treatment option for T2D.^3^

A systematic review and meta-analysis by Liu et al. evaluated the efficacy and safety of IDegLira for T2D, concluding that IDegLira significantly improved glycemic control without increasing the risk of hypoglycemia or weight gain.^2^,^4^ Similarly, IDegLira is associated with more significant reductions in HbA1c, weight loss, and a lower rate of hypoglycemia.^3^ In a real-world setting, IDegLira has been shown to improve time in range and reduce glycemic fluctuations in patients with T2D.^5^ A study by Fadini GP. (2024) highlighted the benefits of IDegLira in reducing HbA1c and body weight in patients with T2D, citing more than one treatment use.^6^

This multicenter study evaluates IDegLira’s effectiveness in the Pakistani T2D population over six months. Analyzing data from various centers, it seeks insights into clinical outcomes, safety, and patient adherence.

## METHODS

This multicenter, retrospective study collected real-world data from various public & private hospitals and primary care clinics in Pakistan. It analyzed medical records of individuals treated with IDegLira alongside basal/premixed insulin, OHAs, or both in the Type-II diabetes (T2D) population. The evaluation occurred at six months (26 weeks), between April 2022 and September 2022.

### Ethical Approval::

The study was approved by the National Bioethics Committee of Pakistan, and the local research ethics committee of Shifa International Hospital (Reference no. 4-87/NBC-738/22/446; dated: February 9, 2022 & reference no. 243-21; dated: July 16, 2021) and complied applicable ICH-GCP guidelines.

Data was collected and eventually analyzed for 183 patients prescribed with IDegLira, administered once daily regardless of meals, as dose steps. One step equals one unit of insulin degludec and 0.036 mg of liraglutide. The maximum dose of IDegLira is 50 steps (50 U insulin degludec and 1.8 mg liraglutide). Patients meeting eligibility criteria (adults ≥18 years with T2D, HbA1c > 7.0%, and who initiated IDegLira at least three months before enrollment) were enrolled for evaluation.

The primary objective was to evaluate IDegLira’s effectiveness in controlling glycemia and the change in HbA1c (%) from baseline to six months in patients with T2D. Secondary objectives included measuring changes in fasting blood sugar (FBS), random blood sugar (RBS), and body weight and safety, i.e., adverse events, including hypoglycemia, during IDegLira treatment. Demographic data is presented as mean (± SD) for continuous variables and count and percentage for categorical measures. Paired t-test was used to determine IDegLira’s impact on variables before and after treatment. P values <0.05 indicated statistical significance. Data was analyzed using Stata version 13.

## RESULTS

### Demographics

Total 183 T2D patients with mean age of 52 ± 10 years were included in this study. Average BMI of 32.6 (±6.0) was calculated using weight & height of all patients. Among all, 102 (55.7%) and 81 (44.3%) participants in this study were males & females, respectively. Of all the patients 62% had hypertension, 48% were obese, 40% had history of dyslipidemia and 7.7% had cardiovascular diseases. Table-I reflects all demographic details of the participants recorded at baseline in this study.

**Table-I:** Demographic Details.

Baseline Characteristics		Mean (±SD), N (%)
Age (in years)		52.4 (±9.8)
Height (in cms)		167.4 (±9.8)
Weight (in Kgs)		90.4 (±16.0)
Body Mass Index (BMI)		32.6 (±6.0)
Heart Rate (BPM)		85.5 (±10.6)
Systolic BP (in mm Hg)		132.8 (±13.9)
Diastolic BP (in mm Hg)		83.4 (±9.4)
Baseline FBS (mmol)		164.8 (±34.8)
Baseline RBS (mmol)		241.5 (±73.8)
Gender	Male	102 (55.7%)
	Female	81 (44.3%)
Hypertension Status	Yes	114 (62.3%)
Obesity	Yes	88 (48.1%)
Cardiovascular Disease	Yes	14 (7.7%)
Dyslipidemia	Yes	74 (40.4%)

### Efficacy outcomes from baseline to the last follow-up:

Significant HbA1c reduction (1.1%) was observed among all the enrolled patients after receipt of six months therapy. The difference in mean HbA1c (%) from the baseline visit to the last follow-up was statistically significant; p-value (<0.01). The outcomes, such as mean FBS and RBS, also showed a marked reduction from baseline to follow-up, with a statistically significant p-value of <0.001 for both. Statistically significant weight reduction, 1.38 ± 3.75 Kgs was also observed amongst enrolled patients (Table-II). On average 23.5 (±7.9) IDegLira units was prescribed to each patient.

**Table-II:** Efficacy Outcomes (HbA1c) from Baseline to Last Follow-up.

	Baseline	Follow-up	
Outcome Measures	Mean (±SD)	Mean (±SD)	P-value
HbA1c	9.5 (±1.8)	8.4 (±1.8)	0.000***
FBS (mg/dl)	164.7 (±36.2)	134.4 (±32.9)	0.000***
RBS (mg/dl)	240.1 (±71.4)	207.6 (±77.8)	0.000***
Weight (Kg)	90.4 (±16.0)	89.2 (±15.7)	0.003***

*Paired T-test of mean difference was used for comparison.

*** =statistically significant.

When observing the changes in HbA1c levels from baseline to follow-up, stratified by medication use (OHAs and/or insulins) at baseline; there was a significant difference in the mean reduction of HbA1c levels among all groups (p<0.001). For those using oral hypoglycemic agents (OHAs), basal insulin, and premixed insulin in adjunct with IDegLira therapy, the mean reduction in HbA1c levels was reported to be 1.1, 1.2, and 0.9, respectively with statistical significance (p<0.001) with exception for RBS change in basal insulin group. Table-III shows the mean reductions of HbA1c, FBS, RBS, and weight after using OHAs or basal or premixed insulin in adjunct with IDegLira therapy with the baseline parameters of using them alone.

**Table-III:** Comparison of HbA1c (%) level and weight changes stratified by the use of oral hypoglycemic agents OHA & Insulins.

OHAs/Insulin	Clinical parameters	Baseline Mean (±SD)	Follow-up Mean (±SD)	P-value
*Oral Hypoglycemic Agents (n=159)*	RBS (mg/dl)	238.5 (±70.2)	208.8 (±80.7)	0.001***
	FBS (mg/dl)	165.5 (±36.3)	135.3 (±33.9)	0.000***
	*HbA1c (%)*	9.5 (±1.8)	8.4 (±1.9)	0.000***
	*Weight (Kg)*	90.7 (±15.9)	89.6 (±15.6)	0.007***
*Basal Insulin (n=24)*	RBS (mmol)	211.4 (±44.2)	190.9 (±50.2)	0.242
	FBS (mmol)	155.9 (±30.8)	124.3 (±19.0)	0.002***
	*HbA1c (%)*	9.4 (±1.3)	8.2 (±1.3)	0.000***
	*Weight (kgs)*	91.3 (±13.4)	89.4 (±13.5)	0.001***
*Premixed Insulin (n=49)*	RBS (mmol)	240.8 (±76.5)	201.1 (±64.8)	0.017***
	FBS (mmol)	157.3 (±26.7)	129.1 (±26.6)	0.005***
	*HbA1c (%)*	9.4 (±1.6)	8.5 (±1.4)	0.000***
	*Weight (kgs)*	92.6 (±17.1)	89.9 (±16.7)	0.000***

*Paired T-test of mean difference was used for comparison.

*** =statistically significant.

### Real World use Assessment:

The study evaluated the reason for starting IDegLira. The most common reason was found to be insufficient efficacy of the previous antidiabetic regime in 118 (64.5%) of the patients, weight gain on the previous antidiabetic regime was found to be the second most common reason for starting IDegLira insulin therapy in n=75(41%) patients. Meanwhile, the high cost of the previous regime, Gastrointestinal (G.I) side effects, injection site reactions, and fear of multiple injections were some of the other reasons for starting IDegLira insulin therapy. Among the total patients (n=183) at follow-up, when asked about experiencing adverse events after using IDegLira for six months, n=32(17%) reported experiencing nausea and n=10(5%) Diarrhea. Only three patients reported hypoglycemia, while other side effects include depression n=3 (2%), fatigue, n=9 (5%), mild anxiety n=2 (1.1%), and headache n=6 (3%).

## DISCUSSION

During the six months follow-up period, patients receiving IDegLira therapy experienced significant improvement in glycemic control and weight loss, yielding benefits in clinical outcomes and patient satisfaction. The statistically significant HbA1c reduction of 1.1% (from 9.5% to 8.4%) is observed in this study (p<0.01), supporting the drug’s efficacy in real-world practice. Our study found that patients using basal insulin with IDeglira had significant HbA1c improvements, unlike those on premixed insulin. The average HbA1c reduction is clinically significant, especially in real-world settings where patient adherence and comorbidities vary widely. In addition, there was a significant decrease in fasting blood glucose (from 164.7 to 134.4 mg/dl) and random blood sugar (from 240.1 to 207.6 mg/dl), further supporting the efficacy of IDegLira in managing blood glucose levels. Improvement in HbA1c levels was seen around the six months follow-up period, which aligns with findings reported in randomized clinical trials.^7^ This consistency with previous research suggests that the impact of treatment on HbA1c levels can be detected within this timeframe. It reinforces the importance of regular monitoring to assess the patient’s response to therapy.^8^

The DUAL II China trial participants, who also received IDegLira, achieved a similar average reduction in HbA1c levels over the same duration.^9^ The DUAL I China trial proved that IDegLira is both effective and well tolerated in the Chinese population with Type-II diabetes (T2D) who fail to achieve the desired glycemic targets with OADs alone.^10^ The study findings indicated that levels of HbA1c on premixed insulin and with a basal bolus had further improvements with IDegLira.^11^ IDegLira significantly reduced FBS and RBS compared to OHAs and insulin (basal/ premixed) alone, showing non-inferiority to these treatments as a single option. Due to the synergistic combination of basal insulin and GLP-1RA (Liraglutide), glycemic control could be achieved using lower doses of IDegLira than with either of the individual treatments alone.^12^,^13^ Liraglutide reduces postprandial glucose increments, contributing to more significant HbA1c reduction with IDegLira compared to degludec.^14^

In a study conducted at a single center in Switzerland, 61 patients switching to IDegLira (mostly from insulin regimens plus OADs experienced a decrease of 1.7% in HbA1c.^15^ FBS was also statistically significantly lower with IDegLira than with degludec treatment alone in a research study, contributing to a lower HbA1c.^16^ This indicates that IDegLira is beneficial for patients with poorly controlled T2D, especially those not achieving optimal results with only basal or premixed insulin and OHAs.^17^,^18^ Obesity significantly increases Type-II diabetes risk, but even modest weight loss can reduce complications.^19^ Our study showed that participants experienced notable weight loss after six months on IDegLira, particularly those using premixed insulin over basal insulin. Significant reduction occurred in individuals taking OADs with either insulin type, likely due to liraglutide’s weight-reducing and insulin-sparing effects.^20^ These findings align with the Italian study, showing decreases in FBG and body weight from baseline to all points.^6^

In real-world practice, IDegLira therapy was initiated due to the insufficient efficacy of available treatments like OHAs and basal insulin and the weight gain associated with these medications. Other factors, such as fear of injections, high treatment costs, gastrointestinal side effects, and hypoglycemia episodes, also contribute to its use.^21^ In our study, 28% of subjects discontinued IDegLira, with 18% citing insufficient efficacy, 16% reporting high costs, 10% due to weight gain, and 8% experiencing gastrointestinal side effects. Notably, fear of multiple injections, hypoglycemia, and injection site reactions were among the least common reasons for discontinuation in our cohort. GI adverse events such as nausea, vomiting, and diarrhea were observed but were infrequent.

These side effects can be minimized by gradually titrating insulin/GLP-1 RA combinations, as recent reports indicate findings.^22^ These results align with the DUAL trials, showing incidences mainly in the first weeks of treatment. Fewer patients using insulin degludec reported adverse GI events than those on liraglutide. In contrast, patients receiving IDegLira displayed an intermediate pattern of GI events, suggesting a potential balancing effect between the two components.^23^ Interestingly, the least reported is the hypoglycemic event, which is evident to a recent study that highlights IDeglira’s effectiveness in metabolic control while reducing the risk of significant hypoglycemia and weight gain.^24^

### Limitations

The large sample study for effective real-world evidence generation can be considered in the future to cater to a large population like Pakistan. Due to real-world nature of the study, no hypoglycemia events were reported. Additionally, this study exclusively involved T2D patients with HbA1c levels exceeding 7.0%. Consequently, it remains uncertain if this approach would be effective for T2D patients with co-morbidities such as high insulin resistance, renal issues, or cardiovascular and ocular complications. Future studies with focus of hypoglycemia follow-up, strict control over parameter ranges and dose titration methods, and better evaluation of effectiveness combination of insulin and liraglutide may be considered.

## CONCLUSION

Real-world data in Pakistan shows that IDegLira is effective for T2D patients with poor glycemic control on basal insulin or premixed insulins with ±OHAs. It achieves good glycemic control with significant weight reduction even at reduced basal insulin doses, making it suitable for primary care settings.
